# Longitudinal mental health outcomes of combat‐injured service members

**DOI:** 10.1002/brb3.2088

**Published:** 2021-03-04

**Authors:** Lauren E. Walker, Jessica Watrous, Eduard Poltavskiy, Jeffrey T. Howard, Jud C. Janak, Warren B. P. Pettey, Lee Ann Zarzabal, Alan Sim, Adi Gundlapalli, Ian J. Stewart

**Affiliations:** ^1^ David Grant USAF Medical Center Travis AFB CA USA; ^2^ Leidos Inc San Diego CA USA; ^3^ University of Texas San Antonio San Antonio TX USA; ^4^ Bexar Data San Antonio TX USA; ^5^ VA Salt Lake City Health Care System Salt Lake City UT USA; ^6^ University of Utah School of Medicine Salt Lake City UT USA; ^7^ PEO Defense Healthcare Management Systems (DHMS) San Antonio TX USA; ^8^ Uniformed Services University of the Health Sciences Bethesda MD USA

**Keywords:** anxiety, depression, injury, military health, post‐traumatic stress disorder, risk factors, veterans

## Abstract

**Background:**

The relationship between traumatic injury and subsequent mental health diagnoses is not well understood and may have significant implications for patient screening and clinical intervention. We sought to determine the adjusted association between traumatic injury and the subsequent development of post‐traumatic stress disorder (PTSD), depression, and anxiety.

**Methods:**

Using Department of Defense and Veterans Affairs datasets between February 2002 and June 2016, we conducted a retrospective cohort study of 7,787 combat‐injured United States service members matched 1:1 to combat‐deployed, uninjured service members. The primary exposure was combat injury versus no combat injury. Outcomes were diagnoses of PTSD, depression, and anxiety, defined by International Classification of Diseases 9th and 10th Revision Clinical Modification codes.

**Results:**

Compared to noninjured service members, injured service members had higher observed incidence rates per 100 person‐years for PTSD (17.1 vs. 5.8), depression (10.4 vs. 5.7), and anxiety (9.1 vs. 4.9). After adjustment, combat‐injured patients were at increased risk of development of PTSD (HR 2.92, 95%CI 2.68–3.17), depression (HR 1.47, 95%CI 1.36–1.58), and anxiety (HR 1.34, 95%CI 1.24–1.45).

**Conclusions:**

Traumatic injury is associated with subsequent development of PTSD, depression, and anxiety. These findings highlight the importance of increased screening, prevention, and intervention in patients with exposure to physical trauma.

## Graphical abstract

In this retrospective cohort study of 7,787 combat‐injured United States service members matched 1:1 to combat‐deployed, uninjured service members, combat‐injured service members had higher incidence rates per 100 person‐years for PTSD (17.1 vs. 5.8), depression (10.4 vs. 5.7), and anxiety (9.1 vs. 4.9) when compared to uninjured service members. After adjustment, combat‐injured patients were at increased risk of development of PTSD (HR 2.92, 95%CI 2.68–3.17), depression (HR 1.47, 95%CI 1.36–1.58), and anxiety (HR 1.34, 95%CI 1.24–1.45). These findings highlight the importance of increased screening, prevention, and intervention in patients with exposure to physical trauma.

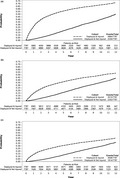

## 1 INTRODUCTION

Since the start of the wars in Iraq and Afghanistan, returning United States (US) veterans have been at high risk for developing mental health conditions (Cameron et al., 2019; Cohen et al., 2010; Hoge et al., 2004; Milliken et al., 2007; Seal et al., 2007). Between 2002 and 2015, more than 708,000 returning veterans seen at the Department of Veterans Affairs (VA) were identified as having a possible mental health outcome and more than 390,000 were diagnosed with post‐traumatic stress disorder (PTSD) (Epidemiology Program, 2015). Another significant concern of the current conflicts is combat injuries (Defense Casualty Analysis System, n.d.). Veterans of the current conflicts have been exposed to mechanisms of combat‐related injury, particularly blasts, that resulted in novel injury patterns (i.e., traumatic brain injury [TBI] and polytrauma) (Hoge et al., 2004; Okie, 2005; U.S. Department of Veterans Affairs, n.d.) that are now survivable due to advances in personal protective equipment, combat casualty care, and medical evacuation (Champion et al., 2003); however, the long‐term impact of these injuries is less clear. A growing body of evidence suggests that when compared to noninjured service members, combat‐injured veterans of recent conflicts may be at heightened risk of subsequent mental health outcomes, including PTSD, depression, and anxiety disorders (Chin & Zeber, 2019; Copeland et al., 2011; Hoge et al., 2006; Howard et al., 2018; Koren et al., 2005; Phillips et al., 2010), although symptoms may not be identified for months or even years after injury (Copeland et al., 2011; Grieger et al., 2006; Seal et al., 2009). Studies of Vietnam veterans have also found increased mental health risk among combat‐injured veterans in the decades following injury (Boscarino, 1995; O’Toole & Catts, 2017). While service members injured in recent conflicts demonstrate high rates of mental health symptoms and diagnoses, including PTSD, depression, and anxiety, in the time periods ranging from the first 2 to 15 months after deployment (Hoge et al., 2006; Koren et al., 2005; Phillips et al., 2010) to up to six years following injury (Chin & Zeber, 2019; Copeland et al., 2011; Howard et al., 2018), less is known about the association between combat injury and mental health in the long‐term.

Research with nonmilitary populations has elucidated the relationship between traumatic injury (i.e., serious physical injury resulting in hospitalization) and mental health. Studies of civilian survivors of other traumatic injuries, such as motor vehicle accidents (Lin et al., 2018; Mayou et al., 1993) and gunshot wounds (Vella et al., 2019) have found that survivors are at heightened mental health risk after injury, with a meta‐analysis of studies on road accident survivors finding a pooled PTSD rate of 22.5% (Lin et al., 2018). Findings in both civilian and military cohorts suggest that further research on long‐term mental health outcomes of survivors of traumatic injury is warranted.

Given the high rate of comorbidities among service members with mental health outcomes, particularly PTSD, and other adverse health outcomes, including hypertension (Cohen et al., 2009; Howard et al., 2018), cardiovascular disease (Dyball et al., 2019; Edmondson & Von Känel, 2017), obesity (Cohen et al., 2009), substance use disorders, (Seal et al., 2011) lower health‐related quality of life (Woodruff et al., 2018), and suicidal ideation (Millner et al., 2019; Ursano et al., ,^2017^, ^2020^) and attempt (Finley et al., 2015; Forehand et al., 2019; O’Donnell et al., 2018), further elucidation of these associations is critical to optimizing prevention and intervention strategies. Our group previously developed a framework hypothesizing that combat injury may lead to poor long‐term outcomes through multiple interacting and overlapping pathways, including changes in health behaviors, mental health, and inflammation (Howard et al., 2018). In order to further explore this model and better understand the independent impact of physical injury on mental health outcomes, we sought to determine the relationship between combat injury and mental health while adjusting for sociodemographics (age, marital status, race/ethnicity, military rank, and military duty status), health behaviors (tobacco use, alcohol abuse/dependence, and opioid abuse/dependence), other diagnoses (hearing injury, insomnia, low‐back pain, and migraine), and TBI severity. The use of these covariates was supported by findings from prior work in service members. For example, previous studies have found differential risk of mental health diagnoses by sociodemographics, including sex, race/ethnicity, and age (Koo et al., 2015; Ramsey et al., 2017; Seal et al., 2009). Substance use disorders, including alcohol use disorder, are prevalent among veterans of recent conflicts and have been associated with up to four times the odds of PTSD and depression (Seal et al., 2011). Opioid abuse (Beyer et al., 2019), chronic pain (Cifu et al., 2013; Higgins et al., 2014; Lee et al., 2019), low‐back pain (Watrous et al., 2020), headache (Jaramillo et al., 2016), and hearing loss (MacGregor et al., 2020) have also been associated with increased likelihood of mental health diagnoses among injured service members. Additionally, injured service members who report poorer sleep and more tobacco use are more likely to screen positive for mental health outcomes (McCabe et al., 2021, 2021). Lastly, TBI severity has been identified as a risk factor for subsequent mental health diagnoses (Chin & Zeber, 2019; Swan et al., 2018).

Many studies of mental health diagnoses after injury in the military population have focused primarily on TBI sustained during combat (Pugh et al., 2016; Swan et al., 2018), and less is known about the relationship between combat injury more broadly and long‐term mental health outcomes of both civilian and military trauma patients. The high utilization of VA health care (Epidemiology Program, 2015) and its continuity of care with the Department of Defense (DoD) health care system provide a unique opportunity to study mental health diagnoses over time while controlling for other covariates. Using a combination of DoD and VA datasets, we randomly selected and matched a cohort of 10,000 combat‐injured service members to combat‐deployed, noninjured service members in order to determine the risk of subsequent development of PTSD, depression, and anxiety. We hypothesized that after adjustment for sociodemographics, health behaviors, other health diagnoses, and TBI severity, history of combat injury would be positively associated with development of these mental health outcomes.

## 2 MATERIALS & METHODS

The David Grant USAF Medical Center Institutional Review Board (IRB), the University of Utah IRB, and the Research Review Committee of the VA Salt Lake City Health Care System reviewed and approved the study protocol. We used DoD and VA datasets to establish two cohorts of current and former US military service members who: 1) were injured during combat operations in Iraq or Afghanistan, and 2) deployed to Iraq or Afghanistan but were not injured. Cohorts were derived by selecting a random sample of 10,000 injured patients from the Department of Defense Trauma Registry (DoDTR) between 1 February 2001 and 14 June 2016 and matching them to uninjured patients in the VA/DoD Identity Repository (VADIR) based on branch of military service (Army, Marine Corps, Navy, Air Force, or Coast Guard), year of birth (± 1 year), and sex. The DoDTR captures demographic, injury, treatment, and outcome information for service members admitted to a Role 3 facility, or surgical field hospital (Joint Trauma System, n.d.). Subjects in the Coast Guard were included with the Navy for this analysis. The uninjured cohort had no injury record in DoDTR or separation from service due to injury. Additional sociodemographics, health characteristics, and mortality data were derived from the Military Health System Management Analysis and Reporting Tool (M2), the Joint VA‐DoD Suicide Data Repository (SDR) National Death Index (NDI) extract (Defense Manpower & Data Center, n.d.), the Defense Manpower Data Center (DMDC), and the Veterans Informatics and Computing Infrastructure (VINCI).

Data on sex, age, rank, branch of service, and Active Duty versus Reserve/Guard status were derived from DoDTR for the combat‐injured cohort and VADIR for the uninjured cohort. Race/ethnicity data were obtained from DMDC unless missing, in which case they were derived from VADIR, or M2 if missing from VADIR. Smoking status, defined by the presence of any smoking behavior throughout the study period, and marital status, as indicated by the closest record to the index date, were obtained from M2. Data on injury type, mechanism of injury, and Injury Severity Score (ISS) were derived from DoDTR. The ISS is a validated, anatomically based scoring system of injury severity with scores ranging from 1–75 (Baker et al., 1974). Injuries with scores ranging from 1–24 were considered mild to moderate; scores ≥ 25 were considered severe. Patients were excluded from analysis if they had any diagnosis PTSD, depression, or anxiety prior to the date of injury (or corresponding index date for the uninjured group). A patient was considered to have the outcome (diagnosis of PTSD, depression, or anxiety) if they had at least two encounters with International Classification of Diseases (ICD) 9th Revision Clinical Modification (CM) or ICD‐10‐CM codes for PTSD, depression (major depression, dysthymia, and other persistent mood disorders), or anxiety disorders (generalized anxiety, panic, phobic, and obsessive‐compulsive disorders), at least 7 days apart, based on previously published methods (Finley et al., 2015). We utilized ICD‐9/10‐CM codes from Armed Forces Health Surveillance Branch (AFHSB) case definitions to define the outcomes (Military Health System, n.d.). Data on traumatic brain injury (TBI) were obtained from M2 and VINCI (for the noninjured cohort), or DoDTR (for the injured cohort). If more than one TBI record was detected in the 60 days following combat injury, only the TBI with the highest severity was used. Although patients in the uninjured group may have had record of TBI, these TBIs were either noncombat TBIs or combat TBIs that were not severe enough for admission to a surgical field hospital. Therefore, no record of combat injury existed for these patients in the DoDTR and they were not grouped with combat‐injured patients. For the uninjured cohort, the first TBI recorded in either M2 or VINCI was used. Using relevant ICD‐9/10‐CM codes, TBI severity was ranked from more to less severe as follows: 1) moderate, severe, or penetrating, 2) mild or unspecified, or 3) no TBI. We also included hearing injury, insomnia, migraine, recurrent low‐back pain, alcohol abuse or dependence, and opioid abuse or dependence as time‐dependent covariates, defined by AFHSB case definition ICD‐9/10 CM diagnosis codes (Military Health System, n.d.). Utilizing previously published methods, we defined recurrent low‐back pain as at least 2 relevant ICD‐9/10‐CM codes at least 90 days apart (Seal et al., 2017); migraine was defined by at least 1 relevant diagnosis code (Cifu et al., 2013); all other covariates were defined by at least 2 relevant diagnosis codes at least 7 days apart (Finley et al., 2015).

The index date was the date of injury in the injured group; in the uninjured group, the index date was the date of injury for the matched patient. If a patient had more than one injury, the index date was the date of first recorded injury. Participants were excluded if they did not have an encounter after the index date, had a diagnosis of the outcomes before the index date, had missing sociodemographic data, or died within 90 days of the index date. The censor date for each patient was the date of last DoD or VA encounter (whichever came last), date of diagnosis of the outcome, end of the study period, or the date of death (whichever came first). Categorical variables are presented as a percentage and compared using a chi‐squared test. Continuous variables are presented as medians and interquartile ranges (IQR) and compared using Wilcoxon rank‐sum test. Incidence rates were compared using Fisher's exact test. Given the different follow‐up between groups and the competing risk of death, we opted to use a time‐to‐event analysis. Specifically, we utilized hazard models for the subdistribution of a competing risk (Fine & Gray, 1999), which compute the hazard ratio (HR), 95% Confidence Interval (CI), and Cumulative Incidence Function, to run stratified models for each outcome with death after 90 days as the competing risk. This included univariate and four nested multivariable models: 1) sociodemographics (age, race/ethnicity, marital status, active duty vs. Reserve/Guard, rank) and injury status (injured vs. uninjured); 2) sociodemographics, injury status, and health behaviors (alcohol abuse and dependence, opioid abuse and dependence, and tobacco use); 3) sociodemographics, injury status, health behaviors, and other health diagnoses (migraine, recurrent low‐back pain, hearing injury, and insomnia); 4) sociodemographics, injury status, health behaviors, other health diagnoses, and TBI severity. Lastly, due to the nonproportionality of injury in the final model, we ran a 5th adjusted model with an interaction term for combat injury in the time periods ≤ 3 years and > 3 years after the index date. Data are presented graphically utilizing cumulative incidence functions. The alpha level was set at .05. All statistical analyses were performed in SAS version 9.4 (SAS Institute, Cary, NC).

## 3 RESULTS

**FIGURE 1:**
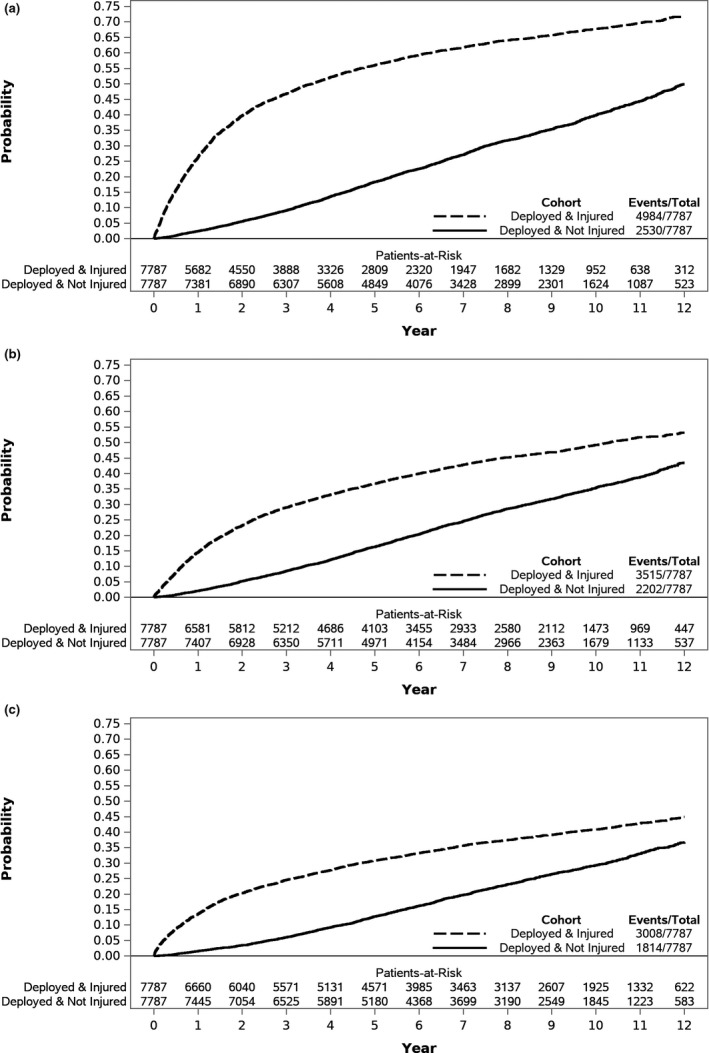
Cumulative Incidence Function for the development of post‐traumatic stress disorder (Panel a), depression (Panel b), and anxiety (Panel c) stratified by injured and uninjured cohorts

Out of 10,000 patients derived from DoDTR, 346 could not be matched to administrative records, leaving 9,654 matched pairs. Of these, 138 were excluded for death within 90 days post index date, 64 for no encounter within the study period, 578 for no encounter after the index date, 1,059 for pre‐existing diagnoses of the outcomes, and 28 for missing variables of interest. In total, 7,787 subjects from each cohort (*N* = 15,574) were included for analysis. A description of the study population is presented in Table 1. Results of the fully adjusted multivariable models for each outcome (Model 4, adjusted for sociodemographics, injury status, health behaviors, other diagnoses, and TBI severity) are presented in Table 2. Supplemental Tables 1, 2 present the results of the univariate, additional nested multivariable models (Model 1, adjusted for sociodemographis and injury status; Model 2, adjusted for sociodemographics, injury status, and health behaviors, and Model 3, adjusted for sociodemographics, injury status, health behaviors, and other diagnoses), and the fully adjusted model with an interaction term for the two time periods after injury (≤3 years and > 3 years; Model 5). Figure 1 presents the Cumulative Incidence Function for each outcome in both groups.

**TABLE 1:** Characteristics of the study population

	Total *N* = 15,574	Combat injured *N* = 7,787	Deployed, not injured *N* = 7,787	*p*‐value
Age, median (IQR)^a^	24 (21–29)	24 (22–28)	24 (21–29)	*
Sex, *N* (%)				*
Male	15,308 (98.3)	7,654 (98.3)	7,654 (98.3)	
Female	266 (1.71)	133 (1.7)	133 (1.7)	
Race/ethnicity, *N* (%)				<.001
Non‐Hispanic White	11,484 (73.7)	5,917 (76.0)	5,567 (71.5)	
Non‐Hispanic Black	1,671 (10.7)	665 (8.5)	1,006 (12.9)	
Hispanic	1,629 (10.5)	822 (10.6)	807 (10.4)	
Asian^b^	530 (3.40)	259 (3.3)	271 (3.5)	
Other/multi‐racial	260 (1.67)	124 (1.6)	136 (1.8)	
Rank, *N* (%)				<.001
Enlisted, Junior (E1–E4)	9,847 (63.2)	4,674 (60.0)	5,173 (66.4)	
Enlisted, Senior (E5–E9)	4,595 (29.5)	2,602 (33.4)	1,993 (25.6)	
Officer	1,132 (7.27)	511 (6.6)	621 (8.0)	
Marital status, *N* (%)				.002
Single	8,377 (53.8)	4,094 (52.6)	4,283 (55.0)	
Married	7,197 (46.2)	3,693 (47.4)	3,504 (45.0)	
Branch of Service, *N* (%)				*
Army	11,352 (72.9)	5,676 (72.9)	5,676 (72.9)	
Air force	256 (1.6)	128 (1.6)	128 (1.6)	
Marines	3,546 (22.8)	1,773 (22.8)	1,773 (22.8)	
Navy	420 (2.7)	210 (2.7)	210 (2.7)	
Duty, *N* (%)				<.001
Active duty	11,646 (74.8)	6,713 (86.2)	4,933 (63.4)	
Reserve/guard	3,928 (25.2)	1,074 (13.8)	2,854 (36.7)	
Total number of days deployed, median (IQR)^f^	340 (207, 500)	302 (164–470)	356 (256–539)	<.001
ISS, median (IQR)	—	6 (2.0 – 13.0)	—	<.001
Injury type, *N* (%)				
Blunt	—	3,822 (49.2)	—	
Penetrating	—	3,749 (48.3)	—	
Burn or other	—	193 (2.5)	—	
Injury mechanism, *N* (%)				<.001
Explosive	—	6,024 (77.5)	—	
Nonexplosive	—	1,745 (22.5)	—	
PTSD, *N* (%)^c^	7,514 (48.3)	4,984 (64.0)	2,530 (32.5)	<.001
Depression, *N* (%)^c^	5,717 (36.7)	3,515 (45.1)	2,202 (28.3)	<.001
Anxiety, *N* (%)^c^	4,822 (31.0)	3,008 (38.6)	1,814 (23.3)	<.001
TBI severity, *N* (%)				<.001
No TBI	8,237 (52.9)	2,057 (26.4)	6,180 (79.4)	
Mild/unclassified	5,085 (32.7)	3,767 (48.4)	1,318 (16.9)	
Moderate/severe^d^	2,252 (14.5)	1,963 (25.2)	289 (3.7)	
Median follow‐up years (IQR)	8.8 (5.6–10.9)	9.1 (5.9–11.1)	8.3 (5.0–10.6)	<.001
Death, *N* (%)^e^	147 (0.9)	80 (0.5)	67 (0.4)	<.001
Incidence rates per 100 person‐years				
PTSD	10.2	17.1	5.8	<.001
Depression	7.8	10.4	5.7	<.001
Anxiety	6.9	9.1	4.9	<.001

Abbreviations: ISS, injury severity score; PTSD, post‐traumatic stress disorder; TBI, traumatic brain injury.

^a^ Years at index date.

^b^ Includes Native Hawaiian and Pacific Islander.

^c^ Incidence after index date.

^d^ Includes penetrating TBI.

^e^ >90 days postindex date

^f^ Calculated for only 6,597 matched pairs due to missing data.

* Matching variable.

**TABLE 2:** Adjusted competing risk models for post‐traumatic stress disorder, depression, and anxiety

	PTSD			Depression			Anxiety		
	HR	95% CI	*p*‐value	HR	95% CI	*p*‐value	HR	95% CI	*p*‐value
Age, years at index date	1.05	0.97–1.13	.223	0.94	0.88–1.01	.078	1.02	0.95–1.09	.655
Married	1.16	1.05–1.29	.004	1.17	1.07–1.29	<.001	1.12	1.01–1.23	.026
Race/ethnicity									
NH White	Ref	—	—	Ref	—	—	Ref	—	—
NH Black	1.14	0.99–1.31	.066	1.22	1.07–1.39	.003	0.86	0.75–0.99	.041
Hispanic	0.99	0.85–1.16	.939	1.09	0.96–1.25	.197	1.04	0.89–1.21	.652
Asian^a^	0.79	0.63–0.99	.041	0.78	0.63–0.98	.029	0.57	0.45–0.73	<.001
Other/multiracial	1.01	0.74–1.39	.933	0.64	0.46–0.89	.008	0.61	0.44–0.86	.005
Rank									
Junior enlisted	Ref	—	—	Ref	—	—	Ref	—	—
Senior enlisted	0.69	0.61–0.78	<.001	0.72	0.64–0.81	<.001	0.78	0.69–0.88	<.001
Officer	0.30	0.24–0.37	<.001	0.32	0.26–0.39	<.001	0.47	0.37–0.59	<.001
Reserve/guard	1.30	1.17–1.46	<.001	1.12	1.01–1.24	.030	0.86	0.77–0.96	.007
Combat injured	2.92	2.68–3.17	<.001	1.47	1.36–1.58	<.001	1.34	1.24–1.45	<.001
Tobacco use									
No	Ref	—	—	Ref	—	—	Ref	—	—
Yes	0.96	0.85–1.09	.524	1.15	1.03–1.29	.012	1.45	1.30–1.62	<.001
Unknown	1.29	1.15–1.44	<.001	1.30	1.17–1.44	<.001	1.15	1.03–1.28	.015
Alcohol abuse/dependence	3.15	2.59–3.84	<.001	2.62	2.24–3.06	<.001	2.04	1.76–2.38	<.001
Opioid abuse/dependence	3.50	1.35–9.09	.010	1.76	1.06–2.92	.028	1.49	1.00–2.23	.053
Hearing injury	1.59	1.40–1.81	<.001	1.20	1.08–1.34	.001	0.98	0.88–1.10	.742
Insomnia	2.52	2.18–2.93	<.001	2.06	1.85–2.31	<.001	2.11	1.89–2.36	<.001
Low‐back pain	2.35	2.09–2.65	<.001	1.90	1.72–2.10	<.001	1.64	1.48–1.81	<.001
Migraine	1.49	1.15–1.95	.003	1.35	1.12–1.63	.002	1.24	1.03–1.49	0.023
TBI severity									
No TBI	Ref	—	—	Ref	—	—	Ref	—	—
Mild/unclassified	2.09	1.88–2.34	<.001	1.69	1.53–1.87	<.001	1.88	1.69–2.08	<.001
Moderate/severe^b^	2.15	1.88–2.47	<.001	1.93	1.69–2.20	<.001	1.95	1.69–2.24	<.001

Abbreviations: NH, non‐Hispanic; PTSD, post‐traumatic stress disorder; TBI, traumatic brain injury.

^a^ Includes Native Hawaiian and Pacific Islander.

^b^ Includes penetrating TBI.

### 3.1 Study population

The study population was predominately male (98.3%) with a median (IQR) age of 24 (21–29) years. The injured cohort was more likely to be non‐Hispanic White (76.0% vs. 71.5%, respectively; *p*<.001) and less likely to be non‐Hispanic Black (8.5% vs. 12.9%, respectively; *p*<.001) than the noninjured cohort. Compared to the uninjured cohort, the injured cohort was more likely to be Senior Enlisted (33.4% vs. 25.6%, respectively), and less likely to be Junior Enlisted (60.0% vs. 66.4%, respectively) or Officer rank (6.6% vs. 8.0%, respectively; *p*<.001). Moderate/severe/penetrating TBI was more common in the injured group (25.2% vs. 3.7%, respectively; *p*<.001). The median ISS in the injured group was 6 (IQR 2.0 – 13.0), with 91.7% of patients with an ISS below 25 (denoting mild to moderate injury severity, as compared to severe injury scores of ≥ 25). Blunt (49.2%) and penetrating (48.3%) injuries were similarly prevalent in the injured group, followed by burn or other injuries (2.5%). Injuries were more likely to be caused by an explosive injury mechanism (77.5%) rather than nonexplosive (22.5%). The uninjured group did not have ISS listed in DoDTR. Compared to noninjured service members, injured service members had higher observed incidence rates per 100 person‐years of postindex date PTSD (17.1 vs. 5.8 per 100 person‐years; *p*<.001), depression (10.4 vs. 5.7 per 100 person‐years; *p*<.001), and anxiety (9.1 vs. 4.9 per 100 person‐years; *p*<.001). The median follow‐up time from the index date for the study population was 8.8 years (IQR 5.6–10.9), with longer follow‐up in the injured (9.1 years, IQR 5.9–11.1) versus uninjured (8.3 years, IQR 5.0–10.6) group.

### 3.2 PTSD

The unadjusted risk of PTSD in the injured cohort was more than four times that in the uninjured cohort (HR 4.25, 95% CI 4.01–4.50; *p*<.001). This risk was not attenuated until the addition of other health diagnoses (hearing injury, insomnia, recurrent low‐back pain, and migraine) in Model 3 (HR 3.92, 95%CI 3.64–4.23; *p*<.001), and was further attenuated after the addition of TBI severity in Model 4 (HR 2.92, 95%CI 2.68–3.17; *p*<.001). The risk for diagnosis of PTSD in the injured group (Figure 1, Panel A) was most pronounced in the first three years following injury (HR 3.37, 95%CI 2.75–4.14; *p*<.001), although the increased risk persisted in the time period more than three years after injury (HR 1.12, 95%CI 1.00–1.25; *p*<.001). In the fully adjusted model, Senior Enlisted (HR 0.69, 95%CI 0.61–0.78; *p*<.001) and Officer rank (HR 0.30, 95%CI 0.24–0.37; *p*<.001) were significantly associated with lower risk of PTSD; alcohol dependence and abuse (HR 3.15, 95%CI 2.59–3.84; *p*<.001), hearing injury (HR 1.59, 95%CI 1.40–1.81; *p*<.001), insomnia (HR 2.52, 95%CI 2.18–2.93; *p*<.001), recurrent low‐back pain (HR 2.35, 95%CI 2.09–2.65; *p*<.001), and mild/unspecified TBI (HR 2.09, 95%CI 1.88–2.34; *p*<.001) or moderate/severe/penetrating TBI (HR 2.15, 95%CI 1.88–2.47; *p*<.001), compared to no TBI, were significantly associated with higher risk of PTSD.

### 3.3 Depression

In the unadjusted model, injury was associated with more than double the risk of development of depression (HR 2.34, 95%CI 2.23–2.46; *p*<.001). The risk was attenuated after the addition of other health diagnoses (HR 1.87, 95%CI 1.75–2.00; *p*<.001), and further attenuated after the addition of TBI severity (HR 1.47, 95%CI 1.36–1.58; *p*<.001). The risk for development of depression in the injured group (Figure 1, Panel B) was highest in the first three years following injury (HR 2.04, 95%CI 1.72–2.66; *p*<.001) compared to the time period more than three years after injury (HR 0.70, 95%CI 0.63–0.79; *p*<.001). In the fully adjusted model, being married (HR 1.17, 95%CI 1.07–1.29; *p*<.001), alcohol abuse or dependence (HR 2.62, 95%CI 2.24–3.06; *p*<.001), insomnia (HR 2.06, 95%CI 1.85–2.31; *p*<.001), recurrent low‐back pain (HR 1.90, 95%CI 1.72–2.10; *p*<.001), mild/unspecified TBI (HR 1.69, 95%CI 1.53–1.87; *p*<.001), and moderate/severe/penetrating TBI (HR 1.93, 95%CI 1.69–2.20; *p*<.001) compared to no TBI were significantly associated with higher risk of depression. Compared to Junior Enlisted rank, Senior Enlisted (HR 0.72, 95%CI 0.64–0.81; *p*<.001), and Officer rank (HR 0.32, 95%CI 0.26–0.39; *p*<.001) were associated with lower risk of depression.

### 3.4 Anxiety

The risk of anxiety associated with combat injury, while attenuated by the addition of covariates, remained significant in each model. Most of the attenuation occurred after the addition of other health diagnoses (HR 1.76, 95%CI 1.65–1.88; *p*<.001) and TBI severity (HR 1.34, 95%CI 1.24–1.45; *p*<.001) compared to the unadjusted model (HR 2.29, 95%CI 2.17–2.42; *p*<.001). The risk of developing anxiety was primarily focused within the first 3 years following injury (HR 3.48, 95%CI 2.67–4.54; *p*<.001), compared to the time period > 3 years after injury (HR 0.65, 95%CI 0.58–0.73; *p*<.001). After adjustment, tobacco use (HR 1.45, 95%CI 1.30–1.62; *p*<.001), alcohol abuse or dependence (HR 2.04, 95%CI 1.76–2.38; *p*<.001), insomnia (HR 2.11, 95%CI 1.89–2.36; *p*<.001), recurrent low‐back pain (HR 1.64, 95%CI 1.48–1.81; *p*<.001), mild/unspecified TBI (HR 1.88, 95%CI 1.69–2.08; *p*<.001), and moderate/severe/penetrating TBI (HR 1.95, 95%CI 1.69–2.24; *p*<.001) compared to no TBI were significantly associated with higher risk of anxiety. Asian (including Native Hawaiian and other Pacific Islander) race/ethnicity was associated with lower adjusted risk of anxiety (HR 0.57, 95%CI 0.45–0.73; *p*<.001) compared to non‐Hispanic White race/ethnicity, as was Senior Enlisted (HR 0.78, 95%CI 0.69–0.88; *p*<.001) and Officer rank (HR 0.47, 95%CI 0.37–0.59; *p*<.001) compared to Junior Enlisted.

## 4 DISCUSSION

We found high incidence rates of PTSD, depression, and anxiety in both combat‐injured (17.1, 10.4, and 9.1 per 100 person‐years, respectively) and noninjured service members (5.8, 5.7, 4.9 per 100 person‐years, respectively). In the fully adjusted models (adjusted for sociodemographics, injury status, health behaviors, diagnoses, and TBI severity), combat‐injured service members were at increased risk for the subsequent development of each outcome, with the risk associated with combat injury concentrated in the first three years following exposure. Although inclusion of other health conditions and TBI severity in the adjusted models attenuated the risk associated with combat injury between 31% and 41%, the associations remained strong and statistically significant. This is the first study to utilize DoD and VA data to demonstrate the adjusted risk trajectories of PTSD, depression, and anxiety over time following combat injury.

Prevalence of postinjury PTSD (64%), depression (45%), and anxiety (39%) in this study was higher than in previous retrospective studies of combat‐injured service members without VA follow‐up, in which 42 (Howard et al., 2018)‐46% (Chin & Zeber, 2019) of injured service members were diagnosed with PTSD, 33% with mood disorders, and 37% with anxiety (Chin & Zeber, 2019). Similarly, incidence of PTSD and depression in this cohort was nearly double that found in a smaller study of combat‐injured veterans in VA care (38% and 27%, respectively) (Copeland et al., 2011). The increased incidence of the outcomes in this cohort highlights the importance of consolidating DoD and VA datasets to facilitate follow‐up of the development of mental health outcomes over time. Combining DoD and VA datasets in this study resulted in a median follow‐up time of 9.1 years (IQR 5.9–11.1) compared to 5.7 (IQR 2.4–9.4) years if only DoD data were used.

In the fully adjusted models, TBI severity was associated with the development of mental health outcomes, consistent with prior work in populations of combat casualties (Chin & Zeber, 2019; Swan et al., 2018). Similar to findings in other studies, recurrent low‐back pain, insomnia, and alcohol abuse/dependence were also associated with increased risk of each of the outcomes after adjustment (Higgins et al., 2014; Seal et al., 2011; Ulmer et al., 2015). Consistent with findings in another large sample of Iraq and Afghanistan veterans in VA care, Asian (including Native Hawaiian and Pacific Islander) veterans were at lower risk of diagnosis for all outcomes when compared to non‐Hispanic White veterans (Koo et al., 2015). Higher military service rank was also associated with lower risk of each outcome. These findings were slightly different from those of a retrospective study in which lower‐ranking service members were at higher risk of subsequent PTSD, but not depression, potentially due to increased combat exposure in their military service roles (Seal et al., 2009).

Our group has previously hypothesized that exposure to combat injury leads to adverse long‐term health outcomes through multiple interacting and overlapping pathways, including mental health diagnoses, health behaviors, such as substance abuse, and inflammation (Howard et al., 2018). Our current findings support an association between injury and subsequent mental health diagnoses and health behaviors, and underscore the complex interplay between these pathways. For example, while alcohol abuse/dependence was associated with mental health outcomes, it is unclear how exactly this health behavior may interact with other mental health symptoms, whether by functioning as a negative coping mechanism for mental health symptoms (e.g., self‐medication hypothesis) (Khantzian, 1997), directly contributing to mental health diagnoses (e.g., alcohol‐induced depressive symptoms) (Boden & Fergusson, 2011), or developing separately following injury. Many health behaviors have evidence‐based therapies available, and, while treatment may not ultimately prevent mental health outcomes, service members should have access to and be encouraged to engage in multidisciplinary care.

Our findings underscore the importance of continued mental health screening following combat injury. Returning service members are screened for mental health symptoms in the postdeployment health assessment (PDHA) immediately following deployment and the postdeployment health reassessment (PDHRA) between 3 and 6 months later. The addition of the PDHRA in 2005 resulted in higher rates of mental health referral, increased reporting of PTSD and depression symptoms, and increased utilization of mental health services when compared to the PDHA alone (Hoge et al., 2006; Milliken et al., 2007). Furthermore, service members who screen positive for depression or PTSD on the PDHRA are more likely to subsequently seek VA care (Vanneman et al., 2017). Our finding that service members are at the highest risk of developing mental health conditions in the first three years after injury further suggests that continued screening in the years following injury may be essential to more comprehensively diagnose and treat mental health conditions in this population. While not completely generalizable, our results suggest that civilian trauma patients who are at risk for long‐term mental health outcomes, such as survivors of gunshot wounds (Vella et al., 2019) and motor vehicle accidents (Lin et al., 2018; Mayou et al., 1993) may also benefit from enhanced mental health screening.

Our work is strengthened by its long follow‐up period and large sample of matched combat casualties. However, it also has limitations. Primarily, our finding that injured service members are particularly at risk during the first three years after injury could be due to increased interaction with the healthcare system, potentially leading to added screening and more opportunities to disclose mental health concerns. Noninjured service members may have experienced, but not reported, mental health symptoms, either due to fewer interactions with the healthcare system or concerns related to seeking treatment (Acosta et al., 2014). However, this limitation was mitigated by ample opportunities for screening and diagnosis within the noninjured group, including both 1) encounters with providers during the first three years following the index date, as well as 2) PDHA and PDHRA screenings postdeployment. Although it is possible that the greater number of encounters within the injured group presented more opportunity to build rapport and potentially disclose concerns to providers, noninjured patients may have had similar opportunity through their own interactions with the healthcare system and screenings following deployment. However, other military service‐specific factors may have disproportionately impacted the noninjured group's willingness to report symptoms. Given service members’ concerns about potential career impact (Acosta et al., 2014; Hom et al., 2017), noninjured service members, who may be more likely to remain in the military postdeployment, may have been less likely to report mental health concerns than their injured counterparts who may have separated or planned to separate from the military.

Due to the use of ICD diagnosis codes, we were unable to capture the full range of mental health symptoms and psychosocial concerns that may not be diagnosed during screening or treatment (Milliken et al., 2007; Seal et al., 2009). Similar limitations exist for the use of ICD codes to define TBI, many of which are mild and may not be diagnosed correctly (Okie, 2005). Additionally, our injured group was defined by patients whose combat injuries resulted in admission to a surgical field hospital; it therefore does not specifically include individuals who sustained nonbattle injuries, combat‐related injuries that did not result in hospital admission, or psychological trauma, although these individuals may have been present in both the injured and uninjured cohorts. While our study data are limited to veterans receiving care at DoD or VA health care systems, this limitation is mitigated by the high utilization of VA health care among Iraq and Afghanistan veterans (Epidemiology Program, 2015). Lastly, important differences exist in mental health outcomes following military service by race/ethnicity, gender (Ramsey et al., 2017), and among gender and sexual minority groups (Mark et al., 2019). Additional work is needed to determine the ways in which combat injury may differentially impact mental health outcomes in these groups.

## 5 CONCLUSION

We found that combat injury is associated with increased risk of developing PTSD, depression, and anxiety after adjustment for sociodemographics, health behaviors, other health diagnoses, and TBI severity. Our findings suggest that continued screening and targeted intervention among injured service members, and potentially civilian survivors of trauma, in the years following injury is warranted.

## 6 DISCLAIMER

The views expressed in this material are those of the authors and do not reflect the official policy or position of the U.S. Government, the Department of Defense, the Department of the Air Force, or the Department of Veterans Affairs.

## CONFLICT OF INTEREST

The authors have no conflicts of interest to report.

## AUTHOR CONTRIBUTIONS

LEW, JW, EP, JTH, JCJ, and IJS: involved in conceptualization; LEW, JW, EP, JTH, JCJ, and IJS: contributed to methodology; EP: involved in formal analysis; IJS and AG: contributed to resources; EP, LAZ, and AS: involved in data curation; LEW: involved in writing—original draft; LEW, JW, EP, JTH, JCJ, WP, LAZ, AS, AG, and IJS: involved in writing—review and editing; IJS: contributed to supervision; IJS and AG: involved in funding acquisition.

### Peer Review

The peer review history for this article is available at https://publons.com/publon/10.1002/brb3.2088.

## Supporting information

Table S1‐S3

## Data Availability

Deidentified data supporting this study may be available from the corresponding author upon request. Additional approvals may be required. The data are not publicly available due to privacy or ethical restrictions.
