# Supplementary material for: Longitudinal mental health outcomes of combat‐injured service members
Source: Brain Behav. 2021 Mar 4;11(5):e02088. doi: 10.1002/brb3.2088 (PMC8119815; doi:10.1002/brb3.2088)
Supplement: Supplementary file 1 — Table S1‐S3 [file BRB3-11-e02088-s001.docx]

Supplemental Table 1. Univariate and competing risk models for post-traumatic stress disorder

|  | Univariate | | | Model 1 | | | Model 2 | | | Model 3 | | | Model 5 | | |
| --- | --- | --- | --- | --- | --- | --- | --- | --- | --- | --- | --- | --- | --- | --- | --- |
|  | HR | 95% CI | P val. | HR | 95% CI | P val. | HR | 95% CI | P val. | HR | 95% CI | P val. | HR | 95% CI | P val. |
| Age^†^ | 0.92 | 0.88-0.96 | <.001 | 1.09 | 1.02-1.17 | 0.016 | 1.12 | 1.04-1.19 | 0.003 | 1.06 | 0.99-1.15 | 0.101 | 1.07 | 0.97-1.19 | 0.155 |
| Married | 1.21 | 1.14-1.28 | <.001 | 1.23 | 1.12-1.35 | <.001 | 1.27 | 1.16-1.40 | <.001 | 1.19 | 1.08-1.32 | <.001 | 1.10 | 0.96-1.26 | 0.175 |
| Race/Ethnicity | | | | | | | | | | | | | | | |
| NH White | Ref | - | - | Ref | - | - | Ref | - | - | Ref | - | - | Ref | - | - |
| NH Black | 0.81 | 0.75-0.89 | <.001 | 1.07 | 0.95-1.22 | 0.276 | 1.09 | 0.96-1.25 | 0.182 | 1.12 | 0.97-1.28 | 0.122 | 1.19 | 0.99-1.43 | 0.063 |
| Hispanic | 1.01 | 0.92-1.10 | 0.871 | 0.97 | 0.84-1.11 | 0.609 | 0.99 | 0.86-1.14 | 0.881 | 0.99 | 0.85-1.15 | 0.883 | 0.97 | 0.79-1.18 | 0.726 |
| Asian^‡^ | 0.77 | 0.66-0.90 | <.001 | 0.73 | 0.59-0.91 | 0.006 | 0.76 | 0.60-0.95 | 0.015 | 0.78 | 0.62-0.98 | 0.032 | 0.79 | 0.59-1.06 | 0.117 |
| Other | 1.06 | 0.85-1.32 | 0.605 | 1.14 | 0.82-1.58 | 0.426 | 1.10 | 0.79-1.53 | 0.561 | 0.99 | 0.73-1.36 | 0.957 | 1.05 | 0.67-1.65 | 0.822 |
| Rank | | | | | | | | | | | | | | | |
| Jr. Enlisted | Ref | - | - | Ref | - | - | Ref | - | - | Ref | - | - | Ref | - | - |
| Sr. Enlisted | 1.01 | 0.93-1.09 | <.001 | 0.65 | 0.58-0.72 | <.001 | 0.70 | 0.62-0.78 | <.001 | 0.66 | 0.58-0.74 | <.001 | 0.80 | 0.67-0.94 | 0.006 |
| Officer | 0.32 | 0.27-0.37 | <.001 | 0.21 | 0.17-0.26 | <.001 | 0.24 | 0.20-0.30 | <.001 | 0.28 | 0.22-0.34 | <.001 | 0.43 | 0.32-0.58 | <.001 |
| Reserve/Guard | 1.54 | 0.50-0.58 | <.001 | 1.06 | 0.96-1.17 | 0.259 | 1.13 | 1.02-1.25 | 0.021 | 1.23 | 1.08-1.32 | <.001 | 1.11 | 0.96-1.29 | 0.174 |
| Injured | 4.25 | 4.01-4.50 | <.001 | 4.61 | 4.31-4.92 | <.001 | 4.86 | 4.52-5.22 | <.001 | 3.92 | 3.64-4.23 | <.001 | - | - | - |
| Tobacco Use | | | | | | | | | | | | | | | |
| No | Ref | - | - |  |  |  | Ref | - | - | Ref | - | - | Ref | - | - |
| Yes | 1.39 | 1.30-1.50 | <.001 |  |  |  | 1.17 | 1.04-1.30 | 0.006 | 1.01 | 0.89-1.13 | 0.919 | 1.18 | 1.01-1.37 | 0.042 |
| Unknown | 1.49 | 1.40-1.60 | <.001 |  |  |  | 1.32 | 1.19-1.46 | <.001 | 1.28 | 1.15-1.43 | <.001 | 1.30 | 1.12-1.50 | <.001 |
| Alcohol^§^ | 3.03 | 2.70-3.40 | <.001 |  |  |  | 3.56 | 2.99-4.23 | <.001 | 3.33 | 2.74-4.03 | <.001 | 2.70 | 2.16-3.38 | <.001 |
| Opioid^§^ | 7.50 | 4.39-12.82 | <.001 |  |  |  | 4.68 | 2.06-10.65 | <.001 | 3.64 | 1.50-8.84 | 0.004 | 2.43 | 0.79-7.44 | 0.121 |
| Hearing injury | 3.78 | 3.46-4.12 | <.001 |  |  |  |  |  |  | 1.72 | 1.52-1.95 | <.001 | 1.59 | 1.38-1.84 | <.001 |
| Insomnia | 5.66 | 5.07-6.33 | <.001 |  |  |  |  |  |  | 2.72 | 2.35-3.14 | <.001 | 2.04 | 1.74-2.40 | <.001 |
| Low-back pain | 2.89 | 2.68-3.13 | <.001 |  |  |  |  |  |  | 2.45 | 2.18-2.76 | <.001 | 1.97 | 1.72-2.25 | <.001 |
| Migraine | 3.00 | 2.58-3.49 | <.001 |  |  |  |  |  |  | 1.64 | 1.27-2.12 | <.001 | 1.24 | 0.96-1.60 | 0.099 |
| TBI Severity | | | | | | | | | | | | | | | |
| No TBI | Ref | - | - |  |  |  |  |  |  |  |  |  | Ref | - | - |
| Mild^¶^ | 4.97 | 4.57-5.41 | <.001 |  |  |  |  |  |  |  |  |  | 1.98 | 1.71-2.29 | <.001 |
| Moderate^††^ | 7.06 | 6.30-7.91 | <.001 |  |  |  |  |  |  |  |  |  | 1.67 | 1.39-2.00 | <.001 |
| Combat Injury*PTSD | | | | | | | | | | | | | | | |
| Injured vs. not injured, >3 years | | | |  |  |  |  |  |  |  |  |  | 1.12 | 1.00-1.25 | 0.061 |
| Injured vs. not injured, ≤3 years | | | |  |  |  |  |  |  |  |  |  | 3.37 | 2.75-4.14 | <.001 |

Abbreviations: NH, non-Hispanic; TBI, traumatic brain injury; CI, Confidence Interval; PTSD, post-traumatic stress disorder; Jr, Junior; Sr, Senior

^†^Years, at index date; ^‡^Includes Native Hawaiian and Pacific Islander; ^§^Abuse/dependence; ^¶^Includes Unclassified TBI; ^††^Includes Severe/Penetrating TBI

Supplemental Table 2. Univariate and competing risk models for depression

|  | Univariate | | | Model 1 | | | Model 2 | | | Model 3 | | | Model 5 | | |
| --- | --- | --- | --- | --- | --- | --- | --- | --- | --- | --- | --- | --- | --- | --- | --- |
|  | HR | 95% CI | P val. | HR | 95% CI | P val. | HR | 95% CI | P val. | HR | 95% CI | P val. | HR | 95% CI | P val. |
| Age^†^ | 0.92 | 0.87-0.97 | <.001 | 1.00 | 0.94-1.07 | 0.930 | 1.00 | 0.94-1.06 | 0.955 | 0.96 | 0.90-1.02 | 0.197 | 0.90 | 0.82-0.99 | 0.027 |
| Married | 1.27 | 1.18-1.35 | <.001 | 1.28 | 1.18-1.38 | <.001 | 1.31 | 1.20-1.43 | <.001 | 1.20 | 1.09-1.31 | <.001 | 1.18 | 1.04-1.34 | 0.010 |
| Race/Ethnicity | | | | | | | | | | | | | | | |
| NH White | Ref | - | - | Ref | - | - | Ref | - | - | Ref | - | - | Ref | - | - |
| NH Black | 0.81 | 0.75-0.89 | <.001 | 1.14 | 1.01-1.27 | 0.028 | 1.16 | 1.03-1.31 | 0.014 | 1.19 | 1.05-1.35 | 0.008 | 1.15 | 0.96-1.38 | 0.130 |
| Hispanic | 1.01 | 0.92-1.10 | 0.871 | 1.05 | 0.93-1.17 | 0.445 | 1.08 | 0.96-1.22 | 0.215 | 1.09 | 0.95-1.24 | 0.213 | 1.08 | 0.91-1.28 | 0.395 |
| Asian^‡^ | 0.77 | 0.66-0.90 | <.001 | 0.71 | 0.58-0.87 | <.001 | 0.76 | 0.62-0.94 | 0.011 | 0.77 | 0.62-0.96 | 0.018 | 0.94 | 0.70-1.27 | 0.690 |
| Other | 1.06 | 0.85-1.32 | 0.605 | 0.73 | 0.54-0.99 | 0.039 | 0.69 | 0.51-0.94 | 0.018 | 0.65 | 0.48-0.89 | 0.007 | 0.92 | 0.59-1.45 | 0.727 |
| Rank | | | | | | | | | | | | | | | |
| Jr. Enlisted | Ref | - | - | Ref | - | - | Ref | - | - | Ref | - | - | Ref | - | - |
| Sr. Enlisted | 0.97 | 0.88-1.06 | 0.501 | 0.72 | 0.65-0.80 | <.001 | 0.70 | 0.63-0.78 | <.001 | 0.76 | 0.66-0.89 | <.001 | 0.68 | 0.61-0.75 | <.001 |
| Officer | 1.07 | 0.98-1.18 | 0.146 | 0.28 | 0.23-0.34 | <.001 | 0.31 | 0.25-0.38 | <.001 | 0.34 | 0.25-0.44 | <.001 | 0.24 | 0.19-0.29 | <.001 |
| Reserve/Guard | 0.75 | 0.63-0.90 | 0.002 | 1.01 | 0.92-1.11 | 0.780 | 1.08 | 0.98-1.19 | 0.127 | 0.97 | 0.85-1.11 | 0.679 | 0.95 | 0.87-1.03 | <.001 |
| Injured | 0.72 | 0.56-0.93 | 0.012 | 2.41 | 2.27-2.56 | <.001 | 1.87 | 1.75-2.00 | <.001 | - | - | - | 2.42 | 2.28-2.56 | <.001 |
| Tobacco Use | | | | | | | | | | | | | | | |
| No | Ref | - | - |  |  |  | Ref | - | - | Ref | - | - | Ref | - | - |
| Yes | 1.47 | 1.36-1.59 | <.001 |  |  |  | 1.29 | 1.17-1.43 | <.001 | 1.17 | 1.05-1.30 | 0.005 | 1.16 | 1.00-1.34 | 0.044 |
| Unknown | 1.58 | 1.46-1.71 | <.001 |  |  |  | 1.33 | 1.21-1.46 | <.001 | 1.29 | 1.16-1.43 | <.001 | 1.56 | 1.01-1.33 | 0.040 |
| Alcohol^§^ | 3.09 | 2.76-3.47 | <.001 |  |  |  | 2.87 | 2.49-3.30 | <.001 | 2.75 | 2.36-3.21 | <.001 | 2.39 | 2.01-2.84 | <.001 |
| Opioid^§^ | 4.77 | 3.24-7.04 | <.001 |  |  |  | 2.39 | 1.50-3.81 | <.001 | 1.83 | 1.12-2.99 | 0.016 | 1.35 | 0.81-2.24 | 0.245 |
| Hearing injury | 2.44 | 2.25-2.64 | <.001 |  |  |  |  |  |  | 1.28 | 1.15-1.42 | <.001 | 1.28 | 1.12-1.45 | <.001 |
| Insomnia | 3.76 | 3.42-4.14 | <.001 |  |  |  |  |  |  | 2.18 | 1.95-2.44 | <.001 | 1.75 | 1.53-2.00 | <.001 |
| Low-back pain | 2.76 | 2.55-3.00 | <.001 |  |  |  |  |  |  | 1.98 | 1.80-2.19 | <.001 | 1.56 | 1.39-1.76 | <.001 |
| Migraine | 2.60 | 2.27-2.98 | <.001 |  |  |  |  |  |  | 1.46 | 1.21-1.75 | <.001 | 1.32 | 1.08-1.61 | 0.007 |
| TBI Severity | | | | | | | | | | | | | | | |
| No TBI | Ref | - | - |  |  |  |  |  |  |  |  |  | Ref | - | - |
| Mild^¶^ | 3.02 | 2.79-3.27 | <.001 |  |  |  |  |  |  |  |  |  | 1.72 | 1.51-1.97 | <.001 |
| Moderate^††^ | 3.76 | 3.39-4.17 | <.001 |  |  |  |  |  |  |  |  |  | 1.51 | 1.26-1.80 | <.001 |
| Combat Injury*Depression | | | | | | | | | | | | | | | |
| Injured vs. not injured, >3 years | | | |  |  |  |  |  |  |  |  |  | 0.70 | 0.63-0.79 | <.001 |
| Injured vs. not injured, ≤3 years | | | |  |  |  |  |  |  |  |  |  | 2.14 | 1.72-2.66 | <.001 |

Abbreviations: NH, non-Hispanic; TBI, traumatic brain injury; CI, Confidence Interval; PTSD, post-traumatic stress disorder; Jr, Junior; Sr, Senior

^†^Years, at index date; ^‡^Includes Native Hawaiian and Pacific Islander; ^§^Abuse/dependence; ^¶^Includes Unclassified TBI; ^††^Includes Severe/Penetrating TBI

Supplemental Table 3. Univariate and competing risk models for anxiety

|  | Univariate | | | Model 1 | | | Model 2 | | | Model 3 | | | Model 5 | | |
| --- | --- | --- | --- | --- | --- | --- | --- | --- | --- | --- | --- | --- | --- | --- | --- |
|  | HR | 95% CI | P val. | 1.04 | .097-1.10 | 0.296 | 1.05 | 0.98-1.12 | 0.140 | 1.03 | 0.96-1.10 | 0.431 | 0.94 | 0.85-1.03 | 0.183 |
| Age^†^ | 0.94 | 0.89-0.99 | 0.022 | 1.25 | 1.15-1.36 | <.001 | 1.24 | 1.14-1.36 | <.001 | 1.15 | 1.05-1.27 | 0.003 | 1.11 | 0.98-1.25 | 0.113 |
| Married | 1.26 | 1.17- 1.35 | <.001 | 1.04 | .097-1.10 | 0.296 | 1.05 | 0.98-1.12 | 0.140 | 1.03 | 0.96-1.10 | 0.431 | 0.94 | 0.85-1.03 | 0.183 |
| Race/Ethnicity | | | | | | | | | | | | | | | |
| NH White | Ref | - | - | Ref | - | - | Ref | - | - | Ref | - | - | Ref | - | - |
| NH Black | 0.74 | 0.67-0.82 | <.001 | 0.85 | 0.75-0.96 | 0.010 | 0.85 | 0.74-0.97 | 0.014 | 0.85 | 0.74-0.98 | 0.024 | 0.86 | 0.72-1.03 | 0.102 |
| Hispanic | 1.02 | 0.91-1.14 | 0.760 | 1.03 | 0.91-1.18 | 0.611 | 1.06 | 0.92-1.21 | 0.442 | 1.02 | 0.88-1.19 | 0.750 | 0.99 | 0.80-1.21 | 0.892 |
| Asian^‡^ | 0.57 | 0.47-0.69 | <.001 | 0.53 | 0.42-0.66 | <.001 | 0.55 | 0.43-0.69 | <.001 | 0.57 | 0.44-0.73 | <.001 | 0.55 | 0.39-0.78 | <.001 |
| Other | 0.78 | 0.59-1.02 | 0.067 | 0.76 | 0.55-1.04 | 0.085 | 0.71 | 0.51-1.00 | 0.050 | 0.65 | 0.47-0.90 | 0.009 | 0.52 | 0.34-0.80 | 0.003 |
| Rank | | | | | | | | | | | | | | | |
| Jr. Enlisted | Ref | - | - | Ref | - | - | Ref | - | - | Ref | - | - | Ref | - | - |
| Sr. Enlisted | 0.98 | 0.90-1.07 | 0.640 | 0.76 | 0.68-0.85 | <.001 | 0.75 | 0.66-0.84 | <.001 | 0.82 | 0.70-0.97 | 0.019 | 0.74 | 0.67-0.83 | <.001 |
| Officer | 0.40 | 0.33-0.48 | <.001 | 0.40 | 0.32-0.49 | <.001 | 0.43 | 0.34-0.54 | <.001 | 0.44 | 0.34-0.58 | <.001 | 0.35 | 0.29-0.43 | <.001 |
| Reserve/Guard | 0.51 | 0.47-0.56 | <.001 | 0.79 | 0.71-0.87 | <.001 | 0.82 | 0.74-0.92 | <.001 | 0.93 | 0.80-1.07 | 0.290 | 0.73 | 0.66-0.80 | <.001 |
| Injured | 2.29 | 2.17-2.42 | <.001 | 2.17 | 2.04-2.30 | <.001 | 1.76 | 1.65-1.88 | <.001 | - | - | - | 2.19 | 2.07-2.32 | <.001 |
| Tobacco Use | | | | | | | | | | | | | | | |
| No | Ref | - | - |  |  |  | Ref | - | - | Ref | - | - | Ref | - | - |
| Yes | 1.80 | 1.65-1.97 | <.001 |  |  |  | 1.61 | 1.45-1.78 | <.001 | 1.49 | 1.34-1.67 | <.001 | 1.44 | 1.24-1.66 | <.001 |
| Unknown | 1.40 | 1.29-1.52 | <.001 |  |  |  | 1.18 | 1.07-1.31 | 0.001 | 1.14 | 1.02-1.27 | 0.018 | 0.97 | 0.84-1.12 | 0.651 |
| Alcohol^§^ | 2.56 | 2.28-2.87 | <.001 |  |  |  | 2.21 | 1.92-2.54 | <.001 | 2.13 | 1.83-2.48 | <.001 | 1.94 | 1.64-2.29 | <.001 |
| Opioid^§^ | 3.28 | 2.39-4.51 | <.001 |  |  |  | 1.92 | 1.34-2.75 | <.001 | 1.56 | 1.04-2.33 | 0.030 | 1.77 | 1.12-2.80 | 0.015 |
| Hearing injury | 2.08 | 1.92-2.25 | <.001 |  |  |  |  |  |  | 1.06 | 0.96-1.18 | 0.257 | 1.00 | 0.88-1.15 | <.958 |
| Insomnia | 3.66 | 3.32-4.03 | <.001 |  |  |  |  |  |  | 2.25 | 2.02-2.51 | <.001 | 1.83 | 1.61-2.09 | <.001 |
| Low-back pain | 2.39 | 2.20-2.59 | <.001 |  |  |  |  |  |  | 1.72 | 1.56-1.90 | <.001 | 1.49 | 1.32-1.69 | <.001 |
| Migraine | 2.29 | 2.00-2.62 | <.001 |  |  |  |  |  |  | 1.32 | 1.10-1.59 | 0.003 | 1.48 | 1.22-1.79 | <.001 |
| TBI Severity | | | | | | | | | | | | | | | |
| No TBI | Ref | - | - |  |  |  |  |  |  |  |  |  | Ref | - | - |
| Mild^¶^ | 3.20 | 2.94-3.48 | <.001 |  |  |  |  |  |  |  |  |  | 1.61 | 1.40-1.84 | <.001 |
| Moderate^††^ | 3.74 | 3.35-4.17 | <.001 |  |  |  |  |  |  |  |  |  | 1.48 | 1.23-1.79 | <.001 |
| Combat Injury*Anxiety | | | | | | | | | | | | | | | |
| Injured vs. not injured, >3 years | | | |  |  |  |  |  |  |  |  |  | 0.65 | 0.58-0.73 | <.001 |
| Injured vs. not injured, ≤3 years | | | |  |  |  |  |  |  |  |  |  | 3.48 | 2.67-4.54 | <.001 |

Abbreviations: NH, non-Hispanic; TBI, traumatic brain injury; CI, Confidence Interval; PTSD, post-traumatic stress disorder; Jr, Junior; Sr, Senior

^†^Years, at index date; ^‡^Includes Native Hawaiian and Pacific Islander; ^§^Abuse/dependence; ^¶^Includes Unclassified TBI; ^††^Includes Severe/Penetrating TBI
